# Heart Rate Responses and Exercise Intensity During A Prolonged 4-Hour Individual Cycling Race among Japanese Recreational Cyclists

**DOI:** 10.3390/sports7050109

**Published:** 2019-05-09

**Authors:** Takashi Nakagata, Shinichiro Murade, Shizuo Katamoto, Hisashi Naito

**Affiliations:** 1 Graduate school of Health and Sports Science, Juntendo University, Hiraka-gakuendai 1-1, Inzai, Chiba 270-1695, Japan; chario1985@yahoo.co.jp (S.M.); skkatasan@gmail.com (S.K.); hnaitou@juntendo.ac.jp (H.N.); 2 Faculty of Health and Sports Science, Juntendo University, Hiraka-gakuendai 1-1, Inzai, Chiba 270-1695, Japan

**Keywords:** cycling race, heart rate monitor, energy cost, energy intake, sports event, aerobic capacity

## Abstract

Heart rate (HR) during different endurance cycling races and events are investigated for professional cyclist, however, enduro races to compete for total laps and distance covered within a fixed time using a circuit course has not yet been investigated. This study examined the heart rate (HR) and exercise intensity during an enduro cycling race. Ten male Japanese amateur cyclists performed cycling individually for at least 2 consecutive hours. HR was measured using an HR monitor during the race, and we estimated the energy expenditure (EE) during the race using the HR–*V*O_2_ relationship in advance. Exercise intensities were defined as percentages of HRmax based on ACSM exercise guideline as follows: moderate intensity, 64–76% HRmax; vigorous intensity, 77–95% HRmax. The HR during the race was 158.9 ± 10.6 bpm (86.4 ± 2.2% HRmax), and exercise intensity is categorized as vigorous intensity. The EE during the race using HR–*V*O_2_ relationship were 12.9 ± 1.2 kcal/kg/hr, which would require a large energy expenditure (EE) during the race. However, energy cost was 0.36 ± 0.04 kcal/kg/km regardless of total distance. The findings indicate that enduro cycling racing is categorized as vigorous intensity (>77% HRmax) for healthy male recreational cyclists though, cycling is an efficient form of transportation.

## 1. Introduction

Cycling exercise is one of the most popular aerobic-type physical activities in a wide range of age groups; walking and jogging have also grown in popularity in recent years as sports. The exercise modality physiological responses of cycling is different in walking and jogging, cycling is a non-weight-bearing exercise, and involves mainly concentric muscle contractions and muscular damage is lower in cycling as compared to jogging [1,2]. Therefore, cycling could be performed in majority populations regardless of age, sex, body mass, and fitness level. Currently, different cycling events and races are held to compete for time or total distance. Endurance races like enduro races to compete for total laps and distance covered within a fixed time using a circuit course are gaining popularity in Japan.

Regarding physiological responses and exercise intensity during different cycling events and races, measurement of heart rate (HR) responses using an automated HR monitor has been investigated [3,4,5,6,7]. For example, Padilla et al. [7] reported that HR during a 5-hour mass-start stage race for international level professional cyclists (all cyclists were members of the same professional racing team and had taken part and finished at least one Tour de France, Giro d’Italia, or Vuelta a Espana) ranged from 119 to 135 bpm (51–61%HRmax), which would require a large energy expenditure (EE) during the race. As such, cyclists in this event have large EE during the 5-hour race. However, to the best of our knowledge, previous studies analyzing the HR and exercise intensity of non-professional (i.e., amateur) and recreational cyclists are limited [8,9,10]. Different cycling events and races are commonly held for recreational cyclists, however, the HR responses during endurance cycling races have not yet been investigated. Exercise intensity of endurance racing could be higher in recreational cyclists than professional cyclists, because recreational cyclists tend to have a lower maximum capacity (e.g., maximum oxygen uptake (*V*O_2_max)). In addition, because cyclists compete for more total laps and distance covered within a fixed time in enduro cycling race individually, our hypothesis is that the HR and exercise intensity during an endurance race of recreational cyclists would be higher intensity.

Thus, this study aimed to determine the exercise intensity of Japanese recreational cyclists during an enduro race. In addition, present study estimated the energy expenditure (EE) during an endure race using HR–*V*O_2_ relationship.

## 2. Materials and Methods

### 2.1. Participants

A total of 28 males aged 24–63 participated in this study. They were recruited according to the following criteria; they perform cycling on the road regularly (more than 2 times/week), and they have at least one year or more of cycling. Participants were excluded from the study if they had coronary heart disease, uncontrolled hypertension, or severe damage to the locomotive organs. Therefore, all participants had no problem with blood pressure and electrocardiogram, and no previous history of established cardiovascular, pulmonary, and neurological diseases. They performed cycling on the road 2–5 times/week, 60–80 km/time, and 120–240 km/week on average. Prior to the study, all participants provided written consent to participate after receiving information on the procedures and purpose of the study. The study protocol was approved by the Research Ethics Review Board of the Juntendo University (22–31), and the study was conducted in accordance with the Declaration of Helsinki for Human Research.

### 2.2. Incremental Exercise Test and HR–VO_2_ Relationship

Participants performed an incremental laboratory cycling test 7 days before the enduro race to measure maximal oxygen consumption (*V*O_2_max) and the HR–*V*O_2_ relationship. The participants in this study refrained from any strenuous physical activity the day before the experiment; fasting (no water restriction) was begun 4 hours before the start of the experiment. Height and body weight were measured before the exercise test, and a graded maximal exercise test (GXT) was performed using the electromagnetic cycle ergometer (PowerMax *V*ll, COMBI WELNESS, Tokyo, Japan). Respiratory gas measurement using indirect calorimetry (AE-300s, Minato Medical Science Company, Ltd.) and a face mask was carried out in our laboratory as previously described [11]. Prior to the start of the experiment, the flow rate sensor was calibrated using a 2 L syringe, and the concentration sensor was calibrated for gas of known concentration (O_2_ 14.98%, CO_2_ 4.99%, N_2_ balance; O_2_ 20.73%, N_2_ balance). All data were processed every 30 sec, and the oxygen uptake (*V*O_2_) and carbon dioxide production (*V*CO_2_) were measured. HR and blood lactate (La) were measured during the exercise test using an automated HR monitor (CS400, Polar, Finland) and lactate analyzer (Lactate scout, EKF Diagnostics, Barleben, Germany). Ratings of perceived exertion (RPE) were recorded using a Borg scale (6–20 steps) [12] during and after the GXT. The GXT started at 1.0 kp for 2 min, then increased to 0.3 kp every 1 min until exhaustion. Participants were asked to maintain the cadence at 60 rpm and to adjust the rhythm with the sound of a metronome during the GXT. We set the following criteria before the GXT to determine the *V*O_2_max: (1) The change in *V*O_2_ of 2.1 ml/kg/min between two consecutive stages was defined as the so-called “plateau or leveling off” during the GXT [13], and the highest maximum value was defined as the *V*O_2_max. If the *V*O_2_ plateau or leveling off was not attained, more than two of the following needed to be met as secondary criteria [14]: (1) maximum HR (HRmax) ≤ 10 beats/min of the age-predicted maximum (age: 220–years old), (2) La ≥ 8 mM/L, and (3) RER ≥ 1.15. All participants met the criteria of *V*O_2_max.

Furthermore, from HR and *V*O_2_ during the GXT in each participant, the relationship between HR and *V*O_2_ was represented by a linear regression equation for each participant. All measurements were carried out in a laboratory where internal atmosphere temperature and humidity were adjusted to 20 °C and 50%, respectively.

### 2.3. Characteristics of the Cycling Race and HR Monitoring

All participants participated in the Motegi enduro cycling race. They cycled around the circuit race individually for 4 hours. The distance of circuit course is 4.8 km, the race course includes uphill sections with a height difference of up to 30.4 m for about 760 m as well as V-shape and 90° turns.

We measured HR during the enduro race using an automated HR monitor (CS400, Polar, Finland) with intervals of 5 seconds. All participants were familiar with the use of HR monitors as they usually trained with them. The EE was estimated using the relationship between HR and *V*O_2_ during GXT in a previous study (during exercise; O_2_ 1 L = 5 kcal) [11].

### 2.4. Statistical Analysis

Microsoft Office Excel 2017 and PASW Statistics version 20.0 (SPSS, IBM Inc. BM Corp., Armonk, NY, USA) were used for data processing and statistical analyses, respectively. All variable results are presented as mean ± SD. Simple linear regression analysis was conducted to examine the relationship between the total distance and HR responses, EE. Participants who cycled individually for at least 2 consecutive hours were included in the analysis, this study included 10 male adults (age, 44.9 ± 12.1 years).

## 3. Results

All participants successfully performed a GXT in laboratory and a 4-hour enduro cycling race individually. Participant characteristics and physiological responses during a GXT is shown in Table 1. Their *V*O_2_max ranged from 49.1 to 60.9 ml/kg/min, and the aerobic capacity level was higher than that reported for the general Japanese.

Physiological responses and energy expenditure during the enduro race are shown in Table 2. The mean values of HR and %HRmax during the race were 158.9 ± 10.6 bpm and 86.4 ± 2.2%HRmax, respectively, resulting in a mean HRmean/HRmax ratio of 0.89 ± 0.03 for the total race period. There was no correlation between total distance and HR response (r = 0.539). The EE during the race calculated by HR–*V*O_2_ relationship were 2881 ± 1038 kcal, 12.9 ± 1.2 kcal/kg/hr and 0.36 ± 0.04 kcal/kg/km. Furthermore, there was no correlation between total distance and EE (kcal/kg/hr and kcal/kg/km).

## 4. Discussion

In a previous study which investigated non-professional (i.e., amateur) cyclists, Neumayr et al. [9] reported in a case report (36-year-old, a well-trained male amateur) that the overall intensity during the ride for 20 hours 51 min was moderate (HRmean = 131 bpm, HRmax = 184 bpm). Similarly, they reported that the mean values of the HRmean and HRmax measured during a cycle-touring event race were 145 and 188 bpm, respectively, for 14 male healthy recreational cyclists (36 ± 6 years old), resulting in a mean HRmean/HRmax ratio of 0.77. Compared to these previous studies for non-professional cyclists, the mean HR measured in the present study during the endure race (158.9 ± 10.6 bpm, 86.4 ± 2.2 %HRmax, and HRmean/HRmax ratio 0.88 ± 0.03) was higher and can be considered as indicating vigorous intensity [15]. Furthermore, the participants cycled at high intensity (>77%HRmax) most of the time from the beginning of the race. The type of endurance race is suggested as the reason for the differences between these results. In general, participants in endurance races compete to receive the highest ranking, and the majority of cyclists perform with supramaximal intensity at the end of the race to break from the pack. The enduro race, however, is a competition of total laps and distance covered within a fixed time using a circuit course; thus, participants may tend to cycle at high intensity from the beginning of the race.

Furthermore, we estimated the EE during the enduro race using their own HR–*V*O_2_ relationship of the GXT, 13.7 ± 1.8 kcal/min and 12.9 ± 1.2 kcal/kg/hr, and they expended 2881 ± 1038 kcal during the enduro race. With regard to exercise intensity of METs, 1 MET is defined as 1kcal/kg/hour, corresponding to an oxygen consumption of 3.5 ml/kg/min [16], the METs of endure race was equivalent to mountain uphill cycling (14 METs) [17]. As such, performance in this enduro race requires large EE and sufficient energy intake to compete during the race. However, energy cost, which represents EE per body weight and distance, was 0.36 ± 0.04 kcal/kg/km on average, this value was lower than the values for general walking (0.5 kcal/kg/km) and jogging (1.0 kcal/kg/km). Furthermore, there was no correlation between total distance and energy cost. This fact indicated that the exercise intensity of the enduro race is vigorous; nevertheless, cycling is a more efficient form of transportation than walking and jogging regardless of total distance. Recently, cycling training has potential to induce muscle hypertrophy in addition to increasing aerobic capacity in young and older age groups [18], therefore, cycling is an alternative exercise/sports for majority of populations who cannot perform walking and jogging with a risk of orthopedic injury, as well as a beneficial exercise to offer a variation for daily physical activity. 

This study was conducted to examine the HR responses of Japanese cyclists during a 4-hour enduro race. The HR responses during the endure race was 158.9 ± 10.6 bpm (86.4 ± 2.2 %HRmax). Our measurements were based on the HR using an automated HR monitor, which is most popular for the measurement of exercise intensity in cycling races. HR monitoring during training and exercise program has become an established means for exercise physiologists, coaches, and athletes to describe exercise and training intensities [19]. Present study was able to compare our results with previously published data gathered using automated HR monitors for competitive professional and/or elite cyclists. However, our study has several limitations. First, the sample of this study was small (n = 10) and all the study participants were males who have a long-term history of fitness and higher fitness level (*V*O_2_max ranged from 49.1 to 60.9 ml/kg/min) as compared to reference interval of the Japanese populations [20]. Previous studies reported that the rate of decline in HRmax was not associated with either sex or physical activity status [21,22], however, a recent study reported that the HR in both sexes is controlled differently, in addition to having different variability using meta-analysis [23]. According to their study, women showed greater vagal activity indexed by high frequency (HF) power of HR variability as compared to men. In addition, Hopker et al. demonstrated that cycling efficiency differs significantly between trained cyclists and recreational cyclists [24]. Therefore, it is important that additional research be done to investigate the physiological responses during cycling races in other population groups including women. Second, we have not considered the drafting effect during the race. In general, cyclists traveling in groups experience a significant reduction in wind resistance, and those behind consume less energy than those in front [25]. Air resistance is proportional to the square of the speed; the cyclist in the front of the group may have a higher HR. Third, the EE during the enduro race was based on the HR–*V*O_2_ relationship. There is a fairly linear relationship between *V*O_2_ and HR at submaximal intensity though, HR responses were influenced by physiological factors (e.g., hydration status, core temperature) and environmental factors [26]. Furthermore, the HR during endurance exercise increases over time, even if it is low–moderate intensity. In addition, the HR during high-intensity exercise was not immediately decreased and maintained a high level at submaximal intensity. Therefore, the EE using HR responses could be overestimated as compared to actual EE during exercise and daily physical activity level [27,28].

## 5. Conclusions

To the best of our knowledge, this is the first study to measure the HR responses of recreational cyclists during an enduro race. The mean HR of endure cycling race was categorized as vigorous intensity (>77% HRmax) for healthy male recreational cyclists, and the enduro race requires a large EE during the race. However, energy cost was 0.36 ± 0.04 kcal/kg/km on average regardless of total distance, cycling is a more efficient form of transportation than walking and jogging. Different types of cycling events and races are held worldwide such as time-trial, ultra-cycling, cycling touring, or mountain bike race, thus it is an interesting challenge to investigate the HR responses and estimate the EE for those cycling events as an area of future research.

## Figures and Tables

**Table 1:** Participant characteristics and physiological responses during a GXT

Number	Age (Years)	Height (cm)	Weight (kg)	BMI (kg/m^2^)	GXT	
					VO_2_max (ml/kg/min)	HRmax (bpm)
1	25	165.5	55.0	20.1	44.7	188
2	31	169.5	58.0	20.2	57.8	187
3	37	175.5	62.5	20.3	60.9	192
4	41	164.6	56.9	21.0	39.3	192
5	42	170.1	62.4	21.6	55.2	184
6	47	179.1	67.5	21.0	53.8	183
7	52	181.0	73.0	22.3	49.1	183
8	52	170.5	69.5	23.9	58.0	187
9	59	175.0	60.0	19.6	51.6	187
10	63	173.2	69.0	23.0	58.4	156
Mean	44.9	172.4	63.4	21.3	52.9	183.9
SD	12.1	5.4	6.1	1.4	6.8	10.3

Note; BMI, body mass index; GXT, graded maximal exercise test.

**Table 2:** Physiological responses and energy expenditure during the enduro race

Number	Distance (km)	HRmean (bpm)	%HRmax (%)	Gross EE (kcal)	EE (kcal/kg/hr)	Energy cost (kcal/km/kg)
1	86.4	169	89.6	1509	12.7	0.32
2	64.8	162	86.7	1632	14.0	0.43
3	151.2	158	82.3	3520	13.8	0.37
4	100.8	171	88.8	1925	10.5	0.34
5	151.2	158	85.7	3433	13.2	0.36
6	158.4	156	85.4	3624	12.7	0.34
7	165.5	156	85.5	3434	11.4	0.28
8	151.2	165	88.4	3851	13.4	0.37
9	72.0	162	86.7	1751	13.5	0.41
10	165.6	133	84.9	4125	14.2	0.36
Mean	126.7	158.9	86.4	2881	12.9	0.36
SD	40.8	10.6	2.2	1038	1.2	0.04
